# On the Absolute Invisibility of Atoms and Molecules

**Published:** 1880-12

**Authors:** A. E. Dolbear


### ON THE ABSOLUTE INVISIBILITY OF ATOMS AND MOLECULES.

BY PROF. A. E. DOLBEAR.

Maxwell gives the diameter of an atom of hydrogen to be such that two million of them in a row would measure a millimeter, but under ordinary physical conditions most atoms are combined with other atoms to form molecules, and such combinations are of all degrees of complexity; thus, a molecule of water contains three atoms, a molecule of alum about one hundred, while molecule of albumen, according to Mulder, contains nine hundred atoms, and there is no reason to suppose albumen to be the most complex of all molecule compounds. When atoms are thus combined it is fair to assume that they are ararnged in three dimensions of space, and that the diameter of the molecule will be approximately as the cube root of the number of atoms it contains, so that a molecule of alum will be equal to

$$(\sqrt[3]{100} = 4.64)\ \frac{4.64}{2000000} = \frac{1}{431000}\ \text{mm.},$$

and a molecule containing a thousand atoms will have a diameter of $\frac{10}{2000000} = \frac{1}{2000000}$ mm. Now, a good microscope will enable a skilled observer to identify an object so small as the $\frac{1}{4000}$ mm. Beale, in his works on the microscopes pictures some fungi as minute as that, and Nobert's test bands and the marking upon the *Amphiplura pellucida*, which are of about the same degree of fineness, are easily resolved by good lenses. If thus the efficiency of the microscope could be increased fifty times ($\frac{20000}{400}=50$) it would be sufficient to enable one to see a molecule of albumen, or if its power could be increased one hundred and seven times it would enable one to see molecule of alum.

Now, Helmholtz has pointed out the probability that interference will limit the visibility of small objects ; but suppose that there should be no difficulty from that source, there are two other conditions which will absolutely prevent us from ever seeing the molecule.

1st. Their motions. A free gaseous molecule of hydrogen at the temperature of 0° C., and a pressure of 760 mm. mercury, has a free path about $\frac{1}{10000}$ mm. in length, its velocity in this free path being 1,860 m, per second, or more than a mile, while its direction of movement is changed million of times per second. Inasmuch as only a glimpse of an object moving no faster than one millimeter per second can be had, for the movements are magnified as well as the object itself, it will be at once seen that a free gaseous molecule can never be seen, not even glimpsed. But suppose such a molecule could be caught and held in the field so it should have no free path. It still has a vibratory motion which constitutes its temperature. The vibratory movement is measured by the number of undulations it sets up in the either per second, and will average five thousand millions of millions, a motion which would make the space occupied by the molecule visibly transparent, that is, it could not be seen. This is true for liquids and solids. Mr. D. N. Hodges finds the path of a molecule of water at its surface to be 0·0000024 mm., and though it is still much less in a solid it must still be much too great for observation.

2d. They are transparent. The rays of the sun stream through the atmosphere, and the latter is not perceptibly heated by them as it would be if absorption took place in it. The air is heated by conduction and contact with the earth, which has absorbed and transformed the energy of the rays. When selective absorption takes place the number of rays absorbed is small when compared with the whole number presented, so that practically the separate molecules would be too transparent to be seen, though their magnitude and motions were not absolute hinderances.

